# Impact of Anatomical Localization on Systemic Inflammatory Markers and Immune Checkpoint CD47 in Desmoid Tumors

**DOI:** 10.3390/jcm15114065

**Published:** 2026-05-24

**Authors:** Sendag Yaslikaya, Suheda Atas Ipek, Ipek Balikci Cicek, Esra Asarkaya, Hatice Asoglu, Muzeyyen Asli Ergozoglu, Mehmet Turker, Yasemin Aydinalp Camadan, Mehmet Mutlu Kidi, Sedat Biter, Tolga Koseci, Kivilcim Eren Ates, Ertugrul Bayram, Gulfiliz Gonlusen, Ismail Oguz Kara

**Affiliations:** 1 Department of Medical Oncology, Giresun Training and Research Hospital, Giresun 28100, Turkey; 2 Department of Medical Oncology, Faculty of Medicine, Cukurova University, Adana 01330, Turkey; suhedaatas92@gmail.com (S.A.I.); easarkaya.91@gmail.com (E.A.); drhatice10@gmail.com (H.A.); a.ergozoglu@gmail.com (M.A.E.); mehmetmutlu_01@hotmail.com (M.M.K.); sedatb23@hotmail.com (S.B.); drtolgakoseci@gmail.com (T.K.); ertugrulbayram84@gmail.com (E.B.); iokara@cu.edu.tr (I.O.K.); 3 Department of Biostatistics and Medical Informatics, Faculty of Medicine, Inonu University, Malatya 44280, Turkey; ipek.balikci@inonu.edu.tr; 4 Department of Medical Oncology, Adana Training and Research Hospital, Adana 01330, Turkey; mmtturker@hotmail.com; 5 Department of Medical Oncology, Sanliurfa Training and Research Hospital, Sanliurfa 63300, Turkey; yaseminaydinalp23@gmail.com; 6 Department of Medical Pathology, Faculty of Medicine, Cukurova University, Adana 01330, Turkey; kivilcimerenates@hotmail.com (K.E.A.); ggonlusen@cu.edu.tr (G.G.)

**Keywords:** desmoid tumor, anatomical localization, systemic inflammation, NLR, PLR, PNI, HALP, PIV, CD47

## Abstract

**Background:** Desmoid tumors (DT) are rare, locally aggressive neoplasms characterized by an unpredictable clinical course. Although anatomical localization has been associated with tumor behavior, its relationship with systemic inflammatory response remains insufficiently explored. This study aimed to evaluate the impact of tumor localization on systemic inflammatory markers and to investigate CD47 expression in DT. **Methods:** This retrospective cohort study included 127 patients diagnosed with DT between 2010 and 2023. Demographic, clinicopathological, and laboratory data were collected. Systemic inflammatory indices, including neutrophil-to-lymphocyte ratio (NLR), platelet-to-lymphocyte ratio (PLR), prognostic nutritional index (PNI), HALP score, and pan-immune-inflammation value (PIV), were calculated. Tumor localization was categorized as trunk, extremity, or head and neck. CD47 expression was evaluated by immunohistochemistry. **Results:** Tumors were most frequently located in the trunk (50.4%), followed by extremities (40.9%) and head and neck region (8.7%). Significant differences in inflammatory markers were observed according to tumor localization. The head and neck group demonstrated lower neutrophil counts (*p* = 0.020), NLR (*p* = 0.009), PLR (*p* < 0.001), and PIV (*p* = 0.003), while showing higher PNI (*p* = 0.043) and HALP scores (*p* = 0.001) compared to trunk-localized tumors. Additionally, smaller tumors (<49 mm) were associated with lower NLR (*p* = 0.041) and neutrophil counts (*p* = 0.015). No detectable CD47 expression was observed in any tumor samples. **Conclusions:** Anatomical localization is closely associated with distinct systemic inflammatory profiles in patients with DT. These findings suggest that tumor location may influence host immune–inflammatory interactions and contribute to the biological heterogeneity of DT. The absence of CD47 expression indicates that alternative immune-related mechanisms may play a role in DT biology. Easily accessible inflammatory markers may provide valuable insights for risk stratification in clinical practice.

## 1. Introduction

Desmoid tumors (DT), also known as aggressive fibromatosis, are rare monoclonal myofibroblastic neoplasms arising from the fascial and musculoaponeurotic structures of deep soft tissues [1,2,3]. Although they exhibit histologically benign features, their most characteristic properties are locally aggressive infiltration into surrounding tissues and an unpredictable clinical course [4,5]. Despite lacking metastatic potential, they may cause significant morbidity by invading adjacent vessels, nerves and vital organs depending on their anatomical location [1,6].

The clinical management of DT remains challenging due to the biological heterogeneity of the tumor [4]. In the literature, tumor size and in particular, anatomical localization (intra-abdominal, trunk or extremity) have been emphasized as key determinants of disease behavior [7,8]. However, these conventional markers are often insufficient to fully capture the unpredictable clinical course of DT, such as its capacity for spontaneous regression or rapid local recurrence despite R0 resection [4]. Furthermore, while molecular markers like CTNNB1 mutations offer deeper insights, their high cost and limited accessibility in routine clinical practice restrict their widespread use [9]. This limitation highlights the need for low-cost, accessible, and objective biomarkers—particularly those reflecting the host’s systemic inflammatory and nutritional state—that may serve as surrogates for tumor biological activity and help characterize different clinical presentations of DT [10]. In this context, evaluating the relationship between systemic inflammatory markers and clinicopathological features may contribute to a better understanding of the biological heterogeneity of DT and the host inflammatory response.

In cancer biology, it is well established that tumors are not solely composed of neoplastic cells but are in continuous interaction with their microenvironment and the host systemic inflammatory response [4]. In addition to markers such as the neutrophil-to-lymphocyte ratio (NLR) and platelet-to-lymphocyte ratio (PLR), which reflect systemic inflammation, indices such as the pan-immune-inflammation value (PIV) and HALP (Hemoglobin, Albumin, Lymphocyte and Platelet) score, which provide a broader assessment of immune and nutritional status, have been shown to reflect biological aggressiveness in many solid tumors [11,12]. Although DT is known to be closely associated with inflammatory pathways, including interferon (IFN) and tumor necrosis factor (TNF) signaling, the specific impact of different anatomical localizations on this systemic inflammatory profile has not been sufficiently investigated in the literature [13,14]. In addition, the CD47 protein, which functions as a “don’t eat me” signal enabling tumor cells to evade immune surveillance, has emerged as an important immune checkpoint in contemporary oncological research [15]. The presence of CD47 expression in DT and its potential association with anatomical localization represent a potential area of investigation for understanding the immunophenotypic characteristics of these tumors. Therefore, this study aimed to determine the impact of anatomical localization on systemic inflammatory response profiles in desmoid tumors and additionally, the evaluation of CD47 expression was planned to further characterize the immunophenotypic features of the tumor.

## 2. Materials and Methods

### 2.1. Study Design and Patient Selection

Ethical approval for this retrospective cohort study was obtained from the institutional ethics committee prior to data collection and analysis (approval number: 56, date: 6 September 2024). The study was conducted in accordance with the principles of the Declaration of Helsinki. In this retrospective cohort study, patients diagnosed with desmoid tumor (DT) at the Department of Oncology of Cukurova University between January 2010 and December 2023 were retrospectively reviewed.

Patients receiving any form of oncological treatment, those with active infections or inflammatory conditions that could affect inflammatory parameters and those with missing data for any of the analyzed inflammatory indices or clinical variables were excluded. Demographic variables (age and sex), tumor localization and tumor size were obtained from medical records and pathology reports.

### 2.2. Hematological, Biochemical and Inflammatory Parameters

Tumor localization was categorized as trunk, extremity, intra-abdominal or head–neck. Maximum tumor diameter and histopathological features were recorded. Peripheral blood parameters obtained within one week prior to surgery or biopsy were retrieved from the hospital database. Systemic inflammatory and nutritional status were evaluated using established indices. The neutrophil-to-lymphocyte ratio (NLR) was calculated as neutrophil count divided by lymphocyte count, and the platelet-to-lymphocyte ratio (PLR) as platelet count divided by lymphocyte count.

The prognostic nutritional index (PNI) was calculated as:

PNI = [10 × serum albumin (g/dL)] + [0.005 × lymphocyte count (/mm^3^)].

The HALP score was calculated as:

HALP = (hemoglobin × albumin × lymphocyte count)/platelet count.

The pan-immune-inflammation value (PIV) was defined as:

PIV = (neutrophil count × platelet count × monocyte count)/lymphocyte count.

Serum albumin and hemoglobin levels were determined using standard biochemical analyses. In addition, the modified Glasgow Prognostic Score (mGPS), based on C-reactive protein (CRP) and serum albumin levels, was also evaluated. Patients with normal CRP levels (≤10 mg/L) were assigned a score of 0. Patients with elevated CRP levels (>10 mg/L) and normal albumin levels (≥3.5 g/dL) were assigned a score of 1, whereas patients with elevated CRP levels (>10 mg/L) and hypoalbuminemia (<3.5 g/dL) were assigned a score of 2.

### 2.3. Immunohistochemical Analysis

For immunohistochemical (IHC) analysis, paraffin-embedded tissue blocks were sectioned at 4 µm and mounted on poly-L-lysine–coated slides. After deparaffinization and rehydration, antigen retrieval was performed in citrate buffer (pH 6.0) using microwave heating. Endogenous peroxidase activity was quenched with 3% hydrogen peroxide. Sections were incubated with a primary anti-CD47 antibody (clone B6H12, dilution 1:200, Abcam, Cambridge, UK) overnight at 4 °C. Immunoreactivity was visualized using the EnVision Detection System (Dako, Glostrup, Denmark), followed by hematoxylin counterstaining. Known CD47-positive breast carcinoma samples were used as external positive controls, whereas sections processed without the primary antibody served as negative controls. Immunohistochemical staining was evaluated independently by two pathologists blinded to the clinical data.

### 2.4. Statistical Analysis

Categorical variables were summarized as counts and percentages. The distribution of continuous variables was assessed using the Shapiro–Wilk test. Depending on distribution, quantitative variables were expressed as mean ± standard deviation or median (minimum–maximum). Group comparisons for categorical variables were performed using the Pearson chi-square test. Continuous variables not conforming to normal distribution were compared using the Kruskal–Wallis test. When overall significance was detected, pairwise comparisons were performed using Bonferroni-adjusted Mann–Whitney U tests. A two-sided *p*-value < 0.05 was considered statistically significant. All analyses were performed using IBM SPSS Statistics for Windows, Version 26.0 (IBM Corp., Armonk, NY, USA).

## 3. Results

**Figure 1:**
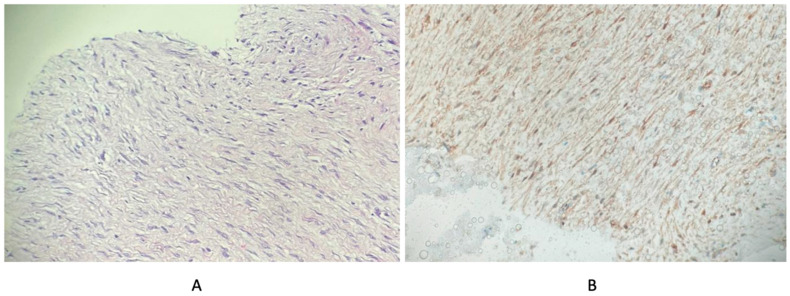
Representative pathological findings of desmoid tumor tissue. (**A**) Hematoxylin and eosin staining demonstrating bland spindle-cell fibroblastic proliferation within a collagenous stroma, consistent with desmoid-type fibromatosis. (**B**) β-catenin immunohistochemistry showing focal nuclear positivity in spindle tumor cells. Original magnification ×200.

A total of 127 patients were included in the study. The median age was 38 years (18–73). Of the patients, 91 (71.7%) were female and 36 (28.3%) were male. Tumors were most frequently located in the trunk (n = 64, 50.4%), and the most common tumor size category was 50–99 mm (n = 55, 43.3%). β-catenin expression was positive in 106 patients (83.1%). Diagnosis was established by surgical excision in 103 patients (82.4%). Demographic, clinicopathological, and treatment characteristics are summarized in Table 1. Representative histopathological and immunohistochemical images of desmoid tumor samples are presented in Figure 1.

The mean mitotic count was 1.56 ± 2.26 and the mean Ki-67 index was 2.06 ± 2.06%. The mean tumor size was 60.55 ± 37.93 mm with a median value of 56 mm (5–170). Hematological and biochemical parameters, together with calculated inflammatory and nutritional indices, are presented in Table 2.

According to tumor location, neutrophil values were significantly lower in the head–neck group compared with the extremity and trunk groups (*p* = 0.020), whereas LY values were higher (*p* = 0.008). Similarly, NLR was lower in the head–neck group than in the trunk group (*p* = 0.009). PLR values were lower in both the head–neck and extremity groups compared with the trunk group (*p* < 0.001), while HALP scores were higher in these groups (*p* = 0.001). In addition, PNI was higher in the head–neck group compared with the trunk group (*p* = 0.043), whereas PIV was lower (*p* = 0.003). Detailed comparisons according to tumor location are presented in Table 3.

When tumor size was evaluated, NE, RDW and NLR were significantly lower in tumors < 49 mm compared with tumors measuring 50–99 mm (*p* = 0.015, *p* = 0.025, and *p* = 0.041, respectively). Other parameters were not significantly associated with tumor size. These findings are shown in Table 4. Tumor size did not differ significantly among anatomical localization groups (head–neck: 54.8 ± 29.6 mm, extremity: 58.9 ± 35.4 mm, trunk: 63.2 ± 40.7 mm; *p* = 0.486).

The distribution of mGPS did not differ significantly according to tumor location (*p* = 0.159) or tumor size (*p* = 0.377) (Table 5).

**Figure 2:**
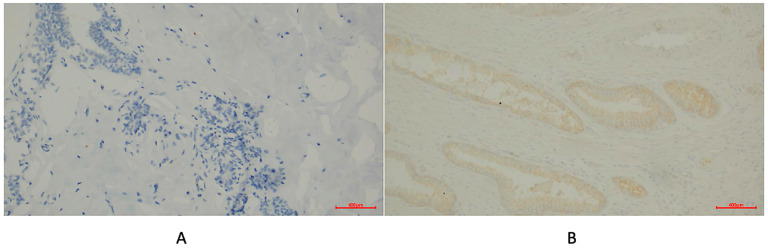
Representative immunohistochemical staining for CD47. (**A**) Desmoid tumor tissue demonstrating absence of CD47 expression. (**B**) Positive control prostate carcinoma tissue showing membranous CD47 positivity. Original magnification ×200.

CD47 IHC showed no detectable expression in desmoid tumor samples. Therefore, no further analyses were performed to assess associations between CD47 expression and clinicopathological variables. Representative immunohistochemical images of desmoid tumor tissue and positive control staining for CD47 are presented in Figure 2.

## 4. Discussion

This study represents one of the comprehensive analyses in the literature demonstrating, with quantitative data, the impact of tumor localization on the systemic inflammatory response in a large cohort of 127 patients with DT. In our cohort, tumor localization was identified in the trunk in 50.4% of cases, in the extremities in 40.9% and in the head and neck region in 8.7%. Immunohistochemical analysis revealed positive nuclear β-catenin expression in 83.1% of patients, consistent with known pathogenetic features reported in the literature [16,17]. Systemic inflammatory indices such as NLR, PLR, HALP score and PIV have previously been associated with tumor aggressiveness, systemic inflammatory burden and adverse clinical outcomes in several solid malignancies [18,19,20]. The key finding of our study is that NLR, PLR, PNI, HALP score and PIV differed significantly according to the anatomical localization of the tumor. According to our analysis, median neutrophil levels in the head and neck group (median: 3200) were significantly lower compared to the extremity (median: 4700) and trunk (median: 4600) groups (*p* = 0.020). These findings indicate that the anatomical compartment in which the tumor develops plays a determining role in shaping the host cellular immune response and systemic inflammatory burden. On the other hand, in the exploratory IHC component of our study, CD47 expression was evaluated; however, no detectable expression was observed in the analyzed tumor samples. Therefore, no further clinicopathological association analyses could be performed.

In the literature, anatomical localization of DT has been reported to be directly associated with biological aggressiveness and recurrence risk [21]. In particular, extra-abdominal tumors (extremity and trunk) have been shown to exhibit significantly worse recurrence-free survival (RFS) compared to abdominal wall tumors (median 31 months vs. not reached, *p* < 0.001) [21]. In addition, intra-abdominal tumors (particularly mesenteric) have been reported to exhibit substantially higher recurrence rates (57–86%) and greater morbidity compared to extra-abdominal or abdominal wall tumors [22,23,24]. Specifically, extremity-localized tumors tend to demonstrate a higher propensity for recurrence due to their aggressive biological behavior, whereas head and neck tumors are considered among the most clinically challenging and potentially life-threatening groups because of their tendency to encase vital neurovascular structures, involve cranial nerves and destroy adjacent bone tissue [25]. The findings from our cohort further support that this difference in clinical aggressiveness is also reflected in systemic inflammatory markers. In our study, NLR (median: 2.62) and PLR (median: 140.95) values were significantly higher in trunk-localized tumors, which are known to have a higher recurrence risk, compared to the head and neck group (NLR: 1.2, *p* = 0.009; PLR: 83.96, *p* < 0.001). This finding is biologically consistent with existing evidence indicating that biomarkers such as NLR and PLR serve as independent predictors of poor prognosis in soft tissue sarcomas and various solid tumors [26,27]. These results suggest that the local invasive potential of the tumor is not solely determined by anatomical boundaries but is also associated with the activation of a systemic inflammatory cascade.

The role of systemic inflammation in the pathogenesis of DT is closely linked to tissue remodeling and inflammatory signaling pathways. Previous studies have demonstrated that tumors arising from different anatomical localizations exhibit distinct gene expression profiles associated with inflammatory pathways, including IFN and TNF signaling; in particular, thoracic DTs have been reported to show higher activation of these pathways compared to abdominal tumors [10,21]. Recent studies have further demonstrated that thoracic (trunk) desmoid tumors exhibit 1.8- to 2.5-fold higher expression of inflammation-related genes, including SOCS1, FOS, MYC, SGK1 and IER3, compared to abdominal tumors [21]. Notably, increased expression of SOCS1, a negative regulator of IFN signaling and FOS, a β-catenin–associated transcription factor, may contribute to the more aggressive biological behavior observed in trunk-localized tumors [28]. In our cohort, PIV values were markedly higher in trunk-localized tumors (median: 412.94) compared to the head and neck group (18.92), representing an approximately 20-fold difference (*p* = 0.003). These findings support the notion that inflammation-related mechanisms may contribute to the biological heterogeneity of DT at the clinical level.

The physiological link between tumor burden and the host systemic response is a well-established phenomenon in mesenchymal neoplasms, in which increasing tumor volume acts as a key driver of pro-inflammatory mediator release, leading to measurable alterations in peripheral blood indices [11,29]. The relationship between tumor size and systemic inflammatory response also represents an important finding of our study. Previous reports have demonstrated that tumors larger than 6 cm are associated with significantly worse recurrence-free survival (*p* = 0.001) [21]. In our cohort, tumor size was also quantitatively associated with inflammatory markers. According to our analysis, NLR was significantly lower in tumors smaller than 49 mm (median: 1.82) compared to those measuring 50–99 mm (median: 2.38) (*p* = 0.041). Similarly, neutrophil counts were lower in the <49 mm group (median: 3800) than in the 50–99 mm group (median: 5200) (*p* = 0.015). Systemic inflammatory markers should not be interpreted as replacements for established clinicopathological factors such as tumor localization or tumor size. Rather, they may provide complementary information by reflecting the host’s inflammatory and nutritional response, which cannot be fully captured by anatomical parameters alone. In this context, the combined evaluation of tumor location, tumor burden and systemic inflammatory indices may offer a more integrated framework for biological characterization and future risk stratification in DT.

## 5. Limitations

The retrospective design of our study and its single-center nature represent the main limitations affecting generalizability. In addition, dynamic changes in inflammatory parameters during tumor regression and progression were not evaluated in this study. On the other hand, treatment approaches according to tumor localization and their association with clinical outcomes were not assessed.

Another limitation is the relatively small sample size of the head–neck subgroup. For this reason, localization-specific subgroup analyses were not comprehensively evaluated in the present study. Nevertheless, the overall cohort size and the broad range of evaluated inflammatory, biochemical, and immunohistochemical parameters strengthen the comprehensiveness and scientific value of the study.

## 6. Conclusions

In conclusion, the present study demonstrated that systemic inflammatory profiles differ according to anatomical localization in patients with desmoid tumors. Significant variations in markers such as NLR, PLR, HALP score, PNI and PIV suggest that anatomical localization may be associated with distinct host inflammatory responses and biological heterogeneity in DT. In addition, certain inflammatory markers were associated with tumor size. These findings may provide complementary biological insight into the inflammatory microenvironment of DT; however, their clinical relevance and relationship with long-term outcomes should be further evaluated in prospective studies.

## Figures and Tables

**Table 1:** Demographic, clinicopathological and treatment characteristics.

Variable	n	%
**Sex**		
Female	91	71.7
Male	36	28.3
**β-catenin expression**		
Negative	21	16.9
Positive	106	83.1
**Tumor location**		
Head and neck	11	8.7
Extremity	52	40.9
Trunk	64	50.4
**Tumor size (mm)**		
<49	52	40.9
50–99	55	43.3
≥100	20	15.7
**Diagnostic method**		
Surgery	105	82.4
Biopsy	22	17.6

**Table 2:** Clinical, laboratory and inflammatory parameters.

Variables	Mean ± SD	Median (Min–Max)
**Pathological parameters**		
Mitotic count	1.56 ± 2.26	1 (0–16)
Ki-67 (%)	2.06 ± 2.06	1 (0–15)
**Clinical parameters**		
Tumor size (mm)	60.55 ± 37.93	56 (5–170)
**Hematological parameters**		
WBC (×10^3^/µL)	8837 ± 3649.34	7850 (3200–21,810)
Neutrophils (/µL)	5607.69 ± 3530.04	4500 (240–19,940)
Lymphocytes (/µL)	2260.44 ± 1011.11	2100 (420–5300)
Monocytes (/µL)	623.98 ± 288.85	600 (100–1700)
Hemoglobin (g/dL)	12.8 ± 1.9	12.7 (7.8–15.4)
RDW (%)	14.54 ± 2.5	13.85 (5.6–25.9)
Platelets (×10^3^/µL)	282.86 ± 102.78	275 (101–746)
PDW (%)	17.43 ± 9.87	16.4 (9.4–63.8)
**Biochemical parameters**		
Albumin (g/dL)	3.73 ± 0.53	3.8 (1.6–4.9)
ALP (U/L)	73.37 ± 27.97	67.5 (28–180)
CRP (mg/L)	10.88 ± 14.78	6 (0–97)
LDH (U/L)	174.35 ± 32.1	170 (126–232)
Triglycerides (mg/dL)	135.92 ± 70.12	102 (46–300)
Glucose (mg/dL)	109.25 ± 33.73	100.5 (67–306)
**Calculated ratios and indices**		
AAR	0.55 ± 0.17	0.56 (0.09–1.04)
CAR	0.31 ± 0.43	0.18 (0–2.69)
TGR	1.20 ± 0.63	1.13 (0.32–3.24)
LAR	4.96 ± 1.55	4.51 (3.34–10.44)
NLR	3.38 ± 4.30	2.12 (0.06–32.93)
PLR	150.47 ± 87.67	128.23 (37–516.67)
HALP score	127.21 ± 1167.81	3.68 (0.68–11,082.89)
PNI	383.75 ± 53.49	391.5 (169–507.5)
PIV (×10^6^)	627.34 ± 975.52	304.93 (2.38–6645.32)

Abbreviations: AAR, albumin-to-alkaline phosphatase ratio; CAR, C-reactive protein to albumin ratio; CRP, C-reactive protein; ALP, alkaline phosphatase; HALP, hemoglobin–albumin–lymphocyte–platelet score; LAR, lymphocyte to albumin ratio; LDH, lactate dehydrogenase; NLR, neutrophil to lymphocyte ratio; PDW, platelet distribution width; PLR, platelet to lymphocyte ratio; PNI, prognostic nutritional index; PIV, pan-immune inflammation value; RDW, red cell distribution width; TGR, triglyceride-to-glucose ratio; WBC, white blood cell count.

**Table 3:** Comparison of hematological, biochemical parameters and calculated indices according to tumor location.

Variables *	Head–Neck	Extremity	Trunk	H Statistic	*p* Value **	Effect Size
WBC (×10^3^/µL)	7.95 (5.48–11.90)	8.30 (3.20–21.81)	7.70 (4.40–20.46)	0.184	0.912	-
NE (/µL)	3200 ^a,b^ (240–8530)	4700 (1200–19,940)	4600 (2300–17,950)	7.812	0.020	0.269 (L)
LY (/µL)	3200 ^b^ (1000–5300)	2400 (500–4850)	1800 (420–4600)	9.679	0.008	0.334 (L)
MO (/µL)	600 (430–1400)	500 (200–1040)	600 (100–1700)	1.666	0.435	-
HGB (g/dL)	12.05 (11–14.8)	13.1 (8.8–17.8)	12.5 (7.8–15.4)	2.635	0.268	-
RDW (%)	13.8 (12.1–17.6)	13.5 (5.6–22.3)	14.1 (12.5–25.9)	3.166	0.205	-
PLT (×10^3^/µL)	314 (101–441)	231 (133–479)	278 (168–746)	4.488	0.106	-
PDW (%)	16.4 (9.9–17.4)	16.5 (9.6–63.8)	16.2 (9.4–62.7)	0.124	0.940	-
Alb (g/dL)	4.0 (3.6–4.4)	3.9 (2.8–4.9)	3.7 (1.6–4.7)	4.639	0.098	-
ALP (U/L)	71.5 (62–123)	68 (56–173)	66 (28–180)	2.935	0.230	-
CRP (mg/L)	2 (0.2–12)	9.5 (0.7–97)	6.85 (0–40)	4.929	0.085	-
LDH (U/L)	174 (137–211)	180 (134–221)	167 (126–232)	0.214	0.898	-
TG (mg/dL)	84 (80–234)	147 (48–300)	99 (46–271)	0.726	0.696	-
Glu (mg/dL)	92.75 (75–138)	99 (67–175)	101 (77–306)	1.140	0.565	-
AAR	0.56 (0.33–0.65)	0.54 (0.21–0.71)	0.57 (0.09–1.04)	1.570	0.456	-
CAR	0.05 (0–0.3)	0.24 (0.02–2.69)	0.24 (0–1.05)	5.757	0.056	-
TGR	0.79 (0.63–1.7)	1.4 (0.4–3.24)	0.94 (0.32–2.46)	1.432	0.489	-
LAR	4.6 (3.34–5.86)	4.49 (3.53–5.94)	4.87 (3.53–10.44)	0.449	0.799	-
NLR	1.2 ^b^ (0.06–3.12)	2.05 (0.48–20.14)	2.62 (1.24–32.93)	9.533	0.009	0.329 (L)
PLR	83.96 ^b^ (37–350)	97.78 ^b^ (44–358)	140.95 (60.22–516.67)	15.614	<0.001	0.538 (L)
HALP score	5.79 ^b^ (1.32–11.3)	5.21 ^b^ (1.25–14.54)	2.93 (0.68–11,082.89)	14.658	0.001	0.505 (L)
PNI	419.75 ^b^ (375–448.5)	394 (292–507.5)	381.5 (169–483)	6.287	0.043	0.217 (L)
PIV (×10^6^)	18.92 ^b^ (23.77–420.00)	304.10 (34.56–5016.22)	412.94 (42.75–6645.32)	11.371	0.003	0.392 (L)

Abbreviations: WBC, white blood cell count; NE, neutrophil; LY, lymphocyte; MO, monocyte; HGB, hemoglobin; RDW, red cell distribution width; PLT, Platelets; PDW, platelet distribution width; Alb, Albumin; ALP, alkaline phosphatase; CRP, C-reactive protein; LDH, lactate dehydrogenase; TG, triglyceride; Glu, glucose; AAR, albumin-to-alkaline phosphatase ratio; CAR, CRP-to-albumin ratio; TGR, triglyceride-to-glucose ratio; LAR, lymphocyte-to-albumin ratio; NLR, neutrophil-to-lymphocyte ratio; PLR, platelet-to-lymphocyte ratio; HALP, hemoglobin–albumin–lymphocyte–platelet score; PNI, prognostic nutritional index; PIV, pan-immune inflammation value; L, large effect size.* Variables are presented as median (minimum–maximum). ** Kruskal–Wallis test. Note: Superscript letters indicate significant post hoc pairwise differences after Bonferroni-adjusted Mann–Whitney U tests. ^a^: significantly different from the extremity group. ^b^: significantly different from the trunk group.

**Table 4:** Comparison of hematological, biochemical parameters and calculated indices according to tumor size.

Variables *	<49 mm	50–99 mm	≥100 mm	H Statistic	*p* Value **	Effect Size
WBC (×10^3^/µL)	7100 (3200–17,800)	8800 (4200–21,810)	7600 (6000–13,630)	5.614	0.060	-
NE (/µL)	3800 ^a^ (240–13,400)	5200 (2100–19,940)	4500 (3400–10,050)	8.336	0.015	0.287 (L)
LY (/µL)	2100 (890–5300)	2000 (420–4900)	2000 (1220–3750)	0.160	0.923	-
MO (/µL)	500 (300–1400)	600 (100–1700)	580 (400–1340)	0.292	0.864	-
HGB (g/dL)	13.3 (7.8–15.4)	11.9 (8.8–15.4)	13.3 (8.8–15.1)	2.997	0.223	-
RDW (%)	13.5 ^a^ (5.6–25.9)	14.4 (11.8–22.3)	14.0 (12.5–16.2)	7.400	0.025	0.255 (L)
Platelets (×10^3^/µL)	281 (133–746)	262 (101–623)	292 (194–526)	0.792	0.673	-
PDW (%)	16.5 (9.4–62.7)	16.5 (9.7–63.8)	15.8 (9.6–17.9)	5.238	0.073	-
Albumin (g/dL)	4.0 (1.6–4.7)	3.75 (2.8–4.9)	3.6 (3.2–4.7)	1.827	0.401	-
ALP (U/L)	68 (28–178)	68 (38–180)	66 (46–102)	0.000	1.000	-
CRP (mg/L)	5 (0.2–40)	11 (0–97)	3 (1–23)	2.674	0.263	-
LDH (U/L)	167 (126–232)	174 (134–221)	164 (148–180)	0.108	0.947	-
TG(mg/dL)	164 (80–300)	98 (46–285)	80 (53–220)	4.597	0.100	-
Glucose (mg/dL)	96.5 (67–306)	98 (75–175)	113.5 (78–187)	2.351	0.309	-
AAR	0.59 (0.09–1.04)	0.53 (0.17–1)	0.53 (0.39–0.74)	1.493	0.474	-
CAR	0.13 (0–1.13)	0.3 (0–2.69)	0.08 (0.03–0.64)	2.276	0.320	-
TGR	1.37 (0.32–2.46)	0.94 (0.4–3.24)	0.66 (0.59–1.18)	3.060	0.217	-
LAR	4.87 (3.34–10.44)	4.28 (3.53–6.87)	4.99 (4.35–5.63)	0.254	0.881	-
NLR	1.82 ^a^ (0.06–9.76)	2.38 (0.48–32.93)	2.15 (1.1–4.59)	6.412	0.041	0.221 (L)
PLR	126.06 (44–441.42)	128.82 (37–516.67)	133.08 (85.33–219.17)	0.671	0.715	-
HALP score	4.2 (0.68–10.72)	3.5 (0.8–11,082.89)	2.81 (1.93–6.03)	0.732	0.693	-
PNI	404.98 (169–478.45)	381.5 (292–507.5)	368.5 (330.95–483)	2.420	0.298	-
PIV (×10^6^)	261.14 (23.77–2599.64)	346.72 (42.75–6645.32)	368.00 (172.55–13,897.45)	3.396	0.183	-

Abbreviations: WBC, white blood cell count; NE, neutrophil; LY, lymphocyte; MO, monocyte; HGB, hemoglobin; RDW, red cell distribution width; PDW, platelet distribution width; ALP, alkaline phosphatase; CRP, C-reactive protein; LDH, lactate dehydrogenase; TG, triglyceride; AAR, albumin-to-alkaline phosphatase ratio; CAR, CRP-to-albumin ratio; TGR, triglyceride-to-glucose ratio; LAR, lymphocyte-to-albumin ratio; NLR, neutrophil-to-lymphocyte ratio; PLR, platelet-to-lymphocyte ratio; HALP, hemoglobin–albumin–lymphocyte–platelet score; PNI, prognostic nutritional index; PIV, pan-immune inflammation value; L, large effect size. * Data are expressed as median (minimum–maximum). ** Kruskal–Wallis test. Note: Superscript letters indicate significant post hoc pairwise differences after Bonferroni-adjusted Mann–Whitney U tests. ^a^: significantly different from the 50–99 mm group.

**Table 5:** Distribution of the Modified Glasgow Prognostic Score (mGPS) according to tumor location and tumor size.

mGPS	Head–Neck	Extremity	Trunk	*p* *	<49 mm	50–99 mm	≥100 mm	*p* *
0	6 (85.71)	10 (41.67)	16 (42.11)	0.159	15 (51.72)	12 (40.00)	5 (50.00)	0.377
1	1 (14.29)	11 (45.83)	13 (34.21)		12 (41.38)	10 (33.33)	3 (30.00)	
2	0 (0.00)	3 (12.50)	9 (23.68)		2 (6.90)	8 (26.67)	2 (20.00)	

* Data are presented as n (%). Pearson chi-square test was used for comparisons.

## Data Availability

The data presented in this study are available on reasonable request from the corresponding author.
